# Spread of *Tst*–Positive *Staphylococcus aureus* Strains Belonging to ST30 Clone among Patients and Healthcare Workers in Two Intensive Care Units

**DOI:** 10.3390/toxins9090270

**Published:** 2017-09-04

**Authors:** Matthaios Papadimitriou-Olivgeris, Eleanna Drougka, Fotini Fligou, Vasiliki Dodou, Fevronia Kolonitsiou, Kriton S. Filos, Evangelos D. Anastassiou, Efthimia Petinaki, Markos Marangos, Iris Spiliopoulou

**Affiliations:** 1 Division of Infectious Diseases, School of Medicine, University of Patras, 26504 Rion-Patras, Greece; papadimitrioumat@hotmail.com (M.P.-O.); marangos@upatras.gr (M.M.); 2 Department of Microbiology, School of Medicine, University of Patras, 26504 Rion-Patras, Greece; eleanna.drougka@gmail.com (E.D.); kolonits@upatras.gr (F.K.); edanastassiou@med.upatras.gr (E.D.A.); 3 National Reference Laboratory for Staphylococci, School of Medicine, University of Patras, 26504 Rion-Patras, Greece; 4 Department of Anaesthesiology and Intensive Care Medicine, University of Patras, 26504 Rion-Patras, Greece; fflig@yahoo.com (F.F.); kritonfilos@yahoo.gr (K.S.F.); 5 Intensive Care Unit, Saint Andrew’s General Hospital, 26335 Patras, Greece; methicu@agandreashosp.gr; 6 Department of Microbiology, School of Medicine, University of Thessaly, Biopolis, 41222 Larissa, Greece; petinaki@uth.gr

**Keywords:** carriage, MRSA, critically ill patients, toxic shock syndrome toxin (TSST-1), Panton–Valentine leukocidin (PVL), MLST, daptomycin, linezolid, vancomycin

## Abstract

*Staphylococcus aureus* is a major cause of infections. Toxic shock syndrome toxin (TSST-1) and Panton–Valentine leukocidin (PVL) are associated with severe clinical syndromes. *S. aureus* colonizing isolates recovered from healthcare workers and patients in the intensive care unit (ICU) of a university hospital comprising Group A were compared with those from adult non-ICU carriers (Group B). *mecA*, *lukS/lukF-PV* (Panton–Valentine leukocidin, PVL), and *tst* (toxic shock syndrome toxin) gene carriage was detected by PCR. Clones were identified in all methicillin-resistant *S. aureus* (MRSA) and toxin-positive methicillin-susceptible strains (MSSA) by pulsed-field gel electrophoresis (PFGE), *agr* groups, and multi locus sequencing typing (MLST). Group A included 90 *S. aureus* isolates, whereas Group B 53. PVL was more frequently found among MRSA vs. MSSA (*p* < 0.001) and in strains of Group B as compared to Group A (*p* < 0.001), consistent with the spread of ST80-IV. Higher incidence of *tst* gene carriage was identified among MSSA vs. MRSA (*P* 0.005) belonging mainly to ST30, and Group A vs. Group B (*P* 0.002). The wide dissemination of ST80-IV mainly in the community is responsible for a high percentage of PVL-positive MRSA, while silent spread of *tst*-positive *S. aureus* clones among ICU patients and personnel poses a threat of hospital transmission and possible severe infections.

## 1. Introduction

*Staphylococcus aureus*—especially methicillin-resistant strains (MRSA)—constitutes a major community and healthcare-associated pathogen worldwide. In Greece, MRSA are frequently isolated from infections [1,2]. *S. aureus* produces a wide range of virulence factors contributing to its pathogenicity and invasiveness, including Panton–Valentine leukocidin (PVL) and staphylococcal toxic shock syndrome toxin (TSST-1), which lead to severe syndromes [2,3].

As in other countries, MRSA epidemiology is related to the health care system (healthcare-associated, HA-MRSA); however, community-associated MRSA (CA-MRSA) isolates have been proved to disseminate among patients since 1998 [2]. In Greece, during the last years, CA-MRSA clones started to infiltrate the health care system, being responsible for nosocomial MRSA infections [2]. Genes encoding PVL (*lukS/lukF-PV*) have been also detected among methicillin-susceptible *S. aureus* (MSSA), but to a lesser extent [2,4,5].

MRSA is a common cause of infections in intensive care units (ICUs). This is due to special patients’ admission processes and the highly susceptible population which renders them the center of antimicrobial-resistant bacteria [1,6]. MRSA search and destroy policies focus on screening patients and healthcare workers (HCWs), defining the groups at risk, isolation of patients, managing outbreaks, carriers’ follow-up after discharge, and elimination of carriage if feasible [7]. Successful implementation has been reported in The Netherlands and the Nordic European countries [7]. In Greece, patients and HCWs have an increased risk of MRSA carriage and infection; thus, screening for MRSA is not only essential for controlling the dissemination of such bacteria that influence patients’ outcome, but is also cost effective [4,7,8]. Although the production of TSST-1 is associated with toxic shock syndrome, *tst*-positive strains usually disseminate silently and do not always cause clinical manifestations [9].

The aim of the present study was to identify MSSA/MRSA colonization among HCWs and adult ICUs’ patients in our university hospital during a surveillance programme, analyze their phenotypes and genotypes (including toxin genes carriage), and compare them with carriage isolates from non-ICU patients, in order to form a basis for future surveillance plans.

## 2. Results

### 2.1. Colonization Results and Studied Isolates

In total, 143 *S. aureus* were obtained and included in the present study (one isolate per person). Among these strains, 57 (40%) were MRSA (35 CA-MRSA and 22 HA-MRSA) (Table 1).

Group A included 90 *S. aureus* isolates recovered from 467 individuals (19% colonization). Twenty-eight strains were MRSA (31%). The epidemiologic prevalence of colonization was 6% (Table 1). From a total of 357 ICU patients, 67 (19%) were *S. aureus* carriers; 22 isolates (33%) were MRSA (epidemiologic prevalence of colonization 6%), whereas 45 (67%) MSSA. Fourteen patients out of 22 MRSA carriers were already colonized upon admission, while the remaining were colonized during hospitalization. From 110 ICU staff screened, 23 (21%) were *S. aureus* carriers; six isolates (26%) were MRSA (epidemiologic prevalence of colonization 6%), and 17 (74%) MSSA. No differences were observed among strains collected from either ICU.

Cohort nursing and infection control practices for MRSA-colonized patients were implemented until three subsequent negative cultures in a weekly basis were obtained. Successful eradication of MRSA among HCWs and patients was achieved by topical mupirocin therapy. No HCW or patient was found MRSA colonized after eradication during the study period or ICU stay, respectively. Two patients developed *S. aureus* bacteraemia by PVL-positive strains: one patient was admitted with bacteraemia caused by MRSA ST80-IV, whereas the other developed bacteraemia during ICU stay by MSSA ST14; each strain was identical to that of colonizing one.

Group B consisted of 53 colonizing *S. aureus* obtained from 161 adult individuals (33% carriage): 29 (55%) isolates were MRSA (epidemiologic prevalence of colonization 18%) and 24 (45%) MSSA.

### 2.2. Susceptibility to Antistaphylococcal Agents

**Figure 1:**
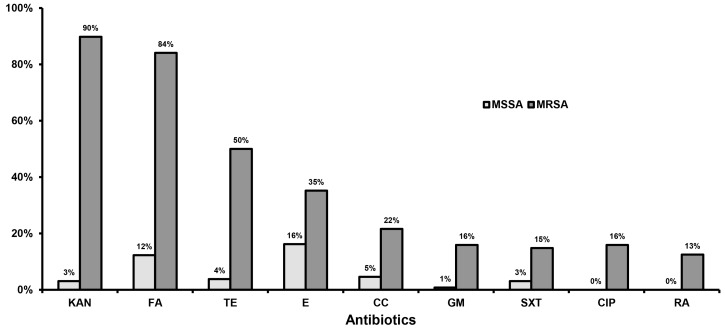
Percentage of resistant isolates among methicillin-susceptible (MSSA) and methicillin-resistant *S. aureus* (MRSA) to antistaphylococcal agents by the disk diffusion method. KAN: kanamycin; FA: fusidic acid; TE: tetracycline; E: erythromycin; CC: clindamycin; GM: gentamicin; SXT: sulphamethoxazole-trimethoprim; CIP: ciprofloxacin; RA: rifampicin.

All MRSA (mecA-positive) were cefoxitin-resistant (disk zone diameter < 21 mm) and oxacillin-resistant (minimum inhibitory concentration (MIC) > 2.0 mg/L). Among MSSA, the MIC of oxacillin ranged from 0.094 to 2.0 mg/L. No statistically significant differences were observed in the antibiotic susceptibility patterns among isolates of both studied groups. On the contrary, MRSA showed significantly higher resistance rates to all antibiotics tested by disk diffusion as compared to MSSA (Figure 1). Moreover, MRSA exhibited statistically significantly higher MIC_90_ to vancomycin (2.0 mg/L vs. 1.5 mg/L), linezolid (1.5 mg/L vs. 1.0 mg/L), and daptomycin (1.0 mg/L vs. 0.19 mg/L) as compared to MSSA, accordingly. All MRSA recovered from ICU patients and HCWs were susceptible to mupirocin (MIC_90_: 0.19 mg/L).

### 2.3. Toxin Genes Carriage

In total, 54 (38%) *S. aureus* carried the PVL genes, whereas 20 (14%) the *tst* toxin gene. No isolate carried both tested toxin genes simultaneously. PVL was significantly more frequently found among total MRSA as compared to MSSA (83% vs. 8%; *p* < 0.001), as well as in Group B strains as compared to Group A (59% vs. 26%; *p* < 0.001). *tst* was significantly more frequent among total MSSA vs. MRSA (20% vs. 4%; *P* 0.005), as well as in Group A as compared to Group B (21% vs. 2%; *P* 0.002) (Table 1).

### 2.4. Clonal Identification

PVL-positive strains (47 MRSA and 7 MSSA) were classified into eight pulsed-field gel electrophoresis (PFGE)/*agr* types, sharing a common one (C/1), whereas, five STs were identified (Table 2). Strains carrying the *tst* gene (2 MRSA and 18 MSSA) were assigned into four PFGE/*agr* types and four STs, sharing one common PFGE/*agr* type, A/3 (Table 2). Type Y/1 was common among MSSA, regardless of toxin genes carriage. Toxin-negative MRSA belonged to four PFGE/*agr* types (three of them common with the toxin-positive MRSA) classified into three STs (Table 2). Toxin-negative MSSA were classified into 16 PFGE/*agr* types sharing five types with the aforementioned strains’ groups (Y/2, C/1, Y/1, C/2, and A/3).

Multi locus sequencing typing (MLST) performed in 38 out of 82 strains (57 MRSA and 25 toxin-positive MSSA) revealed that MRSA ST80-IV clone predominated among six identified ones (Table 2). PVL-positive MRSA belonged to ST80-IV and ST72-IV, while *tst*-positive strains belonged to ST30-IV clone. PVL-positive MSSA were distributed into three STs, while the majority of *tst*-positive MSSA belonged to clone ST30 (10/18 strains or 56%). The remaining eight strains were classified into three additional STs (Table 2).

## 3. Discussion

*S. aureus* is the fifth most frequent pathogen among Greek hospitals [1]. In our settings, 12.5% of total infections and 8.1% of bacteraemias were attributed to *S. aureus* during the study period [10]. The fact that 40% of colonizing *S. aureus* among ICU and non-ICU patients (Groups A and B) were resistant to methicillin underlines their importance to MRSA dissemination. The higher rate of *S. aureus* colonization in Group B (33%)—and especially MRSA (18%) as compared to Group A (19% *S. aureus* and 6% MRSA)—reflects the spread of PVL-positive ST80-IV clone in the community [2]. Although there was a debate for many years concerning the cost-effectiveness of surveillance for MRSA colonization, publications suggest its importance—especially in high-prevalence environments [7]. In 2010, a surveillance programme for MRSA colonization of patients and staff members started at the two adult ICUs of our institution in order to implement interventions to prevent transmission. They included nursing cohorting, contact precautions, reinforcement of hand hygiene policies, and compliance of the staff to infection control practices, combined with decolonization strategies for MRSA-positive patients and HCWs. In total, only 22 ICU patients among 357 tested (6%) were colonized upon admission or during their stay—a percentage comparable to that from international ICUs [5,11]. MRSA colonization among HCWs (6%) proved to be lower than that of the community (18%). Successful eradication and MRSA-positive patients’ cohorting contributed to a restricted number of MRSA infections among ICU patients. Only two patients developed bloodstream infection. One was due to MRSA of ST80-IV clone, whereas the other to MSSA ST14—both colonizing strains, respectively. This fact reinforces the observation published in a previous study showing that the isolate responsible for infection is usually identical to that recovered from nasal cultures, indicating the endogenous origin of such infections [12].

CA-MRSA showed high resistance rates to kanamycin (89%), fusidic acid (89%), tetracycline (53%), and erythromycin (25%), a phenotype related to the ST80-IV clone that is responsible for the majority of infections, especially in the community [2,4]. HA-MRSA showed high percentages of resistance (>20%) to tested antibiotics, but their resistance rates were lower than those reported in a previous Greek study [2]. MSSA were susceptible to most antibiotics, with the exception of erythromycin and fusidic acid (15% and 8% resistance, respectively), which represent the most widely used antibiotics in the community. Layer et al. reported higher resistance rates in German MSSA collections as compared to the present work [11]. The detection of high MICs to vancomycin among MRSA isolates reflects the epidemiology in Greek hospitals highlighting an important therapeutic dilemma, since using vancomycin to treat infections caused by isolates with MICs ≥1.5 mg/L is associated with higher mortality as compared to newer antibiotics with anti-Gram positive action such as linezolid or daptomycin [13,14].

The present study demonstrates a high percentage of PVL-positive *S. aureus* (38%). This is even higher among the MRSA group (83%), particularly in CA-MRSA (97%), among the highest recorded globally due to high dissemination of ST80-IV in the community and the hospital [2,4]. Even though PFGE/*agr* typing revealed higher genetic diversity among PVL-positive strains as compared to MLST, the circulation of specific clones is noteworthy (Table 2). Comparing gene carriage between groups A and B, a statistically significant increased incidence of PVL presence was observed in Group B (59% vs. 26%; *p* < 0.001), reflecting such a dissemination (Table 1 and Table 2).

The percentage of *tst*-positive strains in our collection is low in total (14%), as previously reported [13]. It is lower than those reported from France (60.7%), Spain (51.9%), Germany (40%), or Korea (49.1%) [3,9,11,15]. However, other investigators have reported similar or lower percentages as compared to the present study (Democratic Republic of the Congo: 17.5%, France: 14.3%, Czech Republic: 2%) [16,17,18]. These differences may be partially explained by the dissemination of specific clones, as it was proven in France where the majority of *tst*-positive strains belonged to ST5 [3].

Despite the low percentage of *tst*-positive isolates, the majority (19/20) were recovered from ICU colonized patients and healthcare personnel (Group A: 21% vs. group B: 2%; *P* 0.002). This difference is specifically due to the MSSA prevalence of Group A (27% vs. 4%; *P* 0.018). Peck et al. found similar rates among MSSA and MRSA [15].

No ICU patient was colonized by *tst*-positive *S. aureus* strain upon admission, whereas 14 acquired it during their stay. Observed differences regarding ICU and non-ICU strains are probably due to the dissemination of ST30 clone among patients and ICU staff, since colonized healthcare workers have been responsible for *S. aureus* outbreaks—especially in neonatal ICUs, as reported [19]. This successful spread is reinforced by PFGE/*agr* analysis, which revealed that type A/3 stratified to ST30 and carriage of *tst* gene is consistently present among MRSA since 2001, as well as in MSSA as found in the present study [20]. Clonal complex CC30 (to which ST30 belongs) was proved internationally as a successful one, carrying pathogenicity islands including *tst* gene [21]. In a neonatal ICU in Switzerland, ST30 *tst*-positive *S. aureus* was the predominant clone among colonized neonates (39%) and accounted for 20% of infections [22]. As was shown by Achermann et al., it is possible that exposure to an ICU environment and especially the use of vascular catheters may influence the selection and spread of the specific clone [22]. No patient developed toxic shock syndrome or severe staphylococcal infection, except for the two aforementioned bacteraemias caused by the patients’ colonizing isolates of different genotypes (both PVL-positive). However, this silent dissemination of *tst*-positive isolates combined with the increased mortality observed by infections provoked by such isolates raises the question concerning the utility of regular screening of patients not only for MRSA, but also for toxin-gene carriage [23].

Infection control practices focus on MRSA detection and eradication among HCWs (especially in ICUs), which constitutes a transmission marker of other pathogens, whereas its spread is associated with staff deficit; the impact and risk for patients is also established [7,8]. Our study was performed at a regional level including the Southwestern part of the country, which accounts for one fifth of the country’s population. To the best of our knowledge, no studies analyzing virulence factors of MSSA among other Greek ICU patients were previously performed in order to assess the extent of the dissemination of *tst*-positive ST30 MSSA in Greek hospitals. A limitation in our study is that it was not feasible to identify the carrier and the exact time that *tst*-positive ST30 clone was imported in the ICU, since strains were simultaneously detected among patients and HCWs when the surveillance programme started. These data must be considered in the management of ICU patients, perhaps by using toxin-targeting drugs. Another limitation is the low number of *S. aureus* infections (one MSSA and one MRSA bacteraemia) among patients, which does not permit the evaluation of resistance to antimicrobials and virulence factors in the outcome of such infections.

## 4. Conclusions

The high rate of nasal MRSA colonization among non-ICU patients (18%) in the present study reflects the spread of ST80 clone in the community [2]. Moreover, the lower MRSA carriage level among ICU patients and HCWs (6%) shows that implementation of infection preventive strategies are effective—even in a country with high MRSA prevalence. It is important to understand the factors which contributed to the relatively high percentage of *tst*-positive *S. aureus* strains among ICU patients and HCWs and to clarify if this phenomenon is restricted. Even though no patient revealed the clinical toxic shock syndrome, the risk of silent transmission of *tst*-positive isolates or the horizontal transfer of *tst* gene—especially among MSSA—pose a doubt as to whether screening for MRSA carriage by itself among patients and healthcare personnel is adequate.

## 5. Materials and Methods

### 5.1. Study Design and Hospital

The study was carried out from 1 January 2010 to 31 December 2011 at the University General Hospital of Patras in Greece (UGHP), a tertiary teaching hospital with 700 beds and approximately 100,000 annual admissions (including one-day care facilities). The adult ICU consists of 13 beds with an annual admission rate of 366 patients. Additionally, because of an earthquake, a six bed ICU with 110 admissions per year of “Saint Andrew’s General Hospital” (Patras, Greece), was transferred and hosted in our institution. The two general adult ICUs functioned with their own medical and nursing staff during this period, caring for both medical and surgical patients. Both aforementioned hospitals located in Southwestern Greece serve one fifth of the total population. The study was carried out under the hospital surveillance programme for multi-drug resistant bacterial colonization of patients at risk and HCWs, approved by the Hospital Ethics Committee that waived the need for informed consent (No. 571).

### 5.2. Studied Groups

Patients and recovered isolates were divided into two groups. Group A included *S. aureus* strains isolated during the surveillance programme for MRSA nasal carriage of hospitalized patients and personnel working in both ICUs. In total, 357 ICU patients and 110 ICU staff (including medical, nursing, physiotherapists, and cleaning personnel) were tested for *S. aureus*/MRSA carriage. Only patients with at least three days of ICU stay were included. Samples were collected from anterior nares and pharynx of each ICU patient upon admission and weekly afterwards until discharge, whereas from HCWs every three months. Swabs from each individual were processed as one sample, inoculating them together in 5 mL trypticase soy broth (TSB, BBL, Becton Dickinson, Le Pont de Claix, France); after an overnight incubation, subcultures were performed onto mannitol salt agar plates (BBL, Becton Dickinson) and ChromID MRSA (bioMerieux, La Palme, Les Grottes, France), whereas further incubation was carried out for 24–72 h. In total, 90 *S. aureus* were isolated from this group, consisting of one isolate per patient or HCW. *S. aureus* infections among ICU patients were recorded. No MRSA outbreak was reported in the ICU during the two years prior to start of the study. MRSA-positive HCWs and ICU patients received mupirocin treatment for eradication, and were re-examined for decolonization once weekly for the next three weeks. For MRSA-positive patients, cohort nursing, contact precautions, and reinforcement of hand hygiene policies were applied until successful decolonization.

Group B included 53 colonizing *S. aureus* isolates recovered from 161 currently non-*S. aureus*-infected adult individuals attending outpatients’ facilities (with permission), excluding those previously hospitalized in any ICU during the last two years, as well as household contacts of ICU patients. Nasal swabs from both anterior nares and pharynx were processed as described for Group A.

CA-MRSA strains were defined as those isolated from patients without prior hospitalization the year before sampling, whereas HA-MRSA as those recovered after 48 h of hospitalization or from individuals hospitalized during the last year. MRSA from HCWs were characterized as HA-MRSA because of its high-risk group origin.

### 5.3. S. aureus Identification and Antibiotic Susceptibility Testing

Isolates were identified as *S. aureus* by Gram stain, catalase, and coagulase production (slidex Staph plus test, bioMerieux S.A., Marcy l’Etoile, France) and verified by molecular methods (PCR for 16S rRNA and nuc genes) [24]. Antibiotic susceptibility testing was performed by the disk diffusion method against antistaphylococcal agents: cefoxitin (FOX), tetracycline (TE), rifampicin (RA), gentamicin (GM), kanamycin (KAN), erythromycin (E), clindamycin (CC), ciprofloxacin (CIP), sulphamethoxazole-trimethoprim (SXT) and fusidic acid (FA) [25]. The MICs of oxacillin, mupirocin, vancomycin, linezolid, and daptomycin were determined by Etest (bioMerieux, La Palme, Les Grottes, France) [25].

### 5.4. Polymerase Chain Reaction Procedures

All isolates were tested by PCR for the presence of *mecA*, *lukS/lukF-PV* (PVL) and *tst* (TSST-1) genes [26,27]. Determination of *agr* groups (accessory gene regulator) was performed as described [25,26]. *S. aureus* reference strains ATCC 49775 (PVL-positive) Fri 913 (*tst*-positive), Fri 913 (*agr*-1), Fri 137 (*agr*-2), ATCC 49775 (*agr*-3), and HT 20000195 (*agr*-4) were used as controls. All *mecA*-positive isolates were classified to staphylococcal cassette chromosome mec (SCC*mec*) types by multiplex PCR [28].

### 5.5. Clonal Identification

Clonality was investigated in all *S. aureus* by means of *SmaI* macrorestriction patterns and PFGE. A group of 38 selected isolates among MRSA and toxin-positive MSSA were further typed by multi locus sequencing typing (MLST) [29], on the basis of antibiotic resistance patterns, toxins’ profile, SCC*mec* types, and PFGE/*agr* groups. MRSA clones are named according to their ST-SCC*mec* types.

### 5.6. Statistical Analysis

SPSS statistics version 19.0 (SPSS, Chicago, IL, USA) was used. MICs were compared using the K-sample median-test, while categorical samples were compared with the Fisher’s Exact Test. *p* < 0.05 was considered significant, and values are expressed as percentage.

## Figures and Tables

**Table 1:** Characteristics and statistical analysis of *S. aureus* isolates recovered from two studied groups.

Characteristics	Total (*N* = 143)	^1^ Group A (*N* = 90)			^2^ Group B (*N* = 53)	^3^ *p* Value
		Total (*N* = 90)	ICU Patients (*N* = 67)	ICU Personnel (*N* = 23)		
MRSA	57 (39.9%)	28 (31.1%)	22 (32.8%)	6 (26.1%)	29 (54.7%)	0.008
PVL in total	54 (37.8%)	23 (25.6%)	21 (31.3%)	2 (8.7%)	31 (58.5%)	<0.001
*tst* in total	20 (14%)	19 (21.1%)	14 (20.9%)	5 (21.7%)	1 (1.9%)	0.002
PVL in MSSA	7 (8.1%)	3 (4.8%)	3 (6.7%)	0 (0%)	4 (16.7%)	0.091
*tst* in MSSA	18 (20.1%)	17 (27.4%)	14 (31.1%)	3 (17.6%)	1 (4.2%)	0.018
PVL in MRSA	47 (82.5%)	20 (71.4%)	18 (81.8%)	2 (33.3%)	27 (93.1%)	0.041
*tst* in MRSA	2 (3.5%)	2 (7.1%)	0 (0%)	2 (33.3%)	0 (0%)	0.237
CA-MRSA in MRSA	35 (61.4%)	8 (28.6%)	8 (36.4%)	0 (0%)	27 (93.1%)	<0.001
PVL in CA-MRSA	34 (97.1%)	8 (100%)	8 (100%)	0 (0%)	26 (96.3%)	>0.999
PVL in HA-MRSA	13 (59.1%)	12 (60%)	10 (71.4%)	2 (33.3%)	1 (50%)	0.560
*tst* in CA-MRSA	0 (0%)	0 (0%)	0 (0%)	0 (0%)	0 (0%)	-
*tst* in HA-MRSA	2 (9.1%)	2 (10%)	0 (0%)	2 (33.3%)	0 (0%)	>0.999

^1^ Group A: colonizing isolates from patients and health care workers (HCWs) in intensive care units (ICUs); ^2^ Group B: colonizing isolates from non-ICU patients; ^3^ Comparison between Group A and Group B total values. MRSA: methicillin-resistant *Staphylococcus aureus*; CA-MRSA: community-associated MRSA; HA-MRSA: healthcare-associated MRSA; MSSA: methicillin-susceptible *S. aureus;* PVL: Panton–Valentine leucocidin; *tst*: gene coding for staphylococcal toxic shock syndrome toxin.

**Table 2:** Clones in relation to toxin gene carriage among *S. aureus* recovered from both studied groups. Numbers of isolates are given in parentheses.

	^1^ Clones	PFGE Type/*agr* Allele	CA-/HA-MRSA	^2^ Group A (*N* = 90)		^3^ Group B (*N* = 53)
				ICU Patients (*N* = 67)	ICU Personnel (*N* = 23)	
PVL-positive MRSA (47)	ST80-IV (45)	C/*3* (43), C/*1* (1), C/NT (1)	33/12	16	2	27
	ST72-IV (2)	B/*1* (1), E/*1* (1)	0/2	2	0	0
PVL-positive MSSA (7)	ST14 (4)	Y/*1* (2), A/*1* (1), C/*2* (1)	-	3	0	1
	ST97 (1)	C/*1* (1)	-	0	0	1
	ST101 (2)	C/*2* (2)	-	0	0	2
*tst*-positive MRSA (2)	ST30-IV (2)	A/*3* (2)	0/2	0	2	0
*tst*-positive MSSA (18)	ST30 (10)	A/*3* (9), A/*4* (1)	-	7	2	1
	ST2123 (6)	Y/*1* (6)	-	6	0	0
	ST27 (1)	Y/*2* (1)	-	1	0	0
	ST45 (1)	F/*1* (1)	-	0	1	0
Toxin negative MRSA (8)	SΤ239-III (4)	B/*1* (3), B/NT (1)	0/4	3	0	1
	ST225–II (2)	C/*3* (2)	0/2	1	1	0
	ST30–IV (2)	A/*3* (2)	1/1	0	1	1
Toxin negative MSSA (61)	-	16 PFGE/*agr* types	-	28	14	19

PFGE: pulsed field gel electrophoresis; CA-MRSA: community-associated MRSA; HA-MRSA: healthcare-associated MRSA; NT: non-typable to *agr* allele. ^1^ Clones: clones defined by multi locus sequencing typing (MLST) among MSSA and MLST-SCC*mec* types among MRSA; ^2^ Group A: colonizing isolates from patients and healthcare workers in ICUs; ^3^ Group B: colonizing isolates from non-ICU patients during the study period.
